# Supplementary material for: Multi-omics reveals changed energy metabolism of liver and muscle by caffeine after mice swimming
Source: PeerJ. 2024 Jan 3;12:e16677. doi: 10.7717/peerj.16677 (PMC10771084; doi:10.7717/peerj.16677)
Supplement: Supplemental Information 8 [file peerj-12-16677-s008.pdf]

METHODS

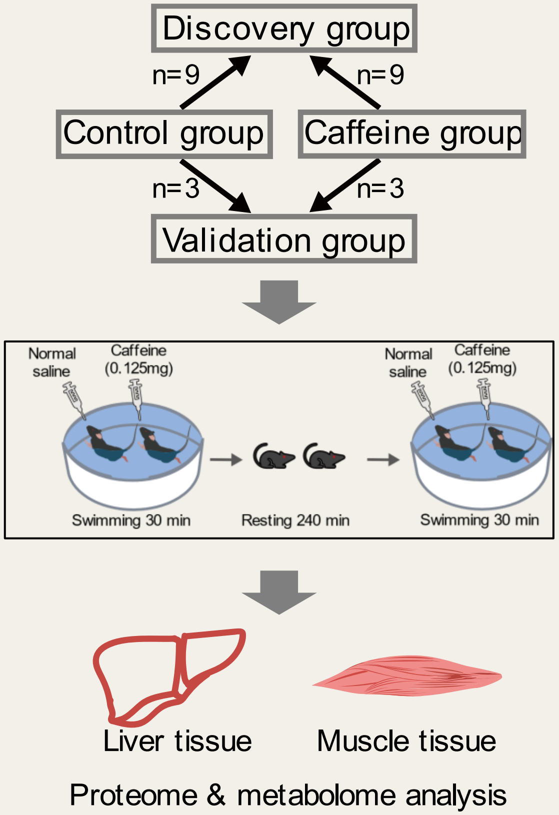

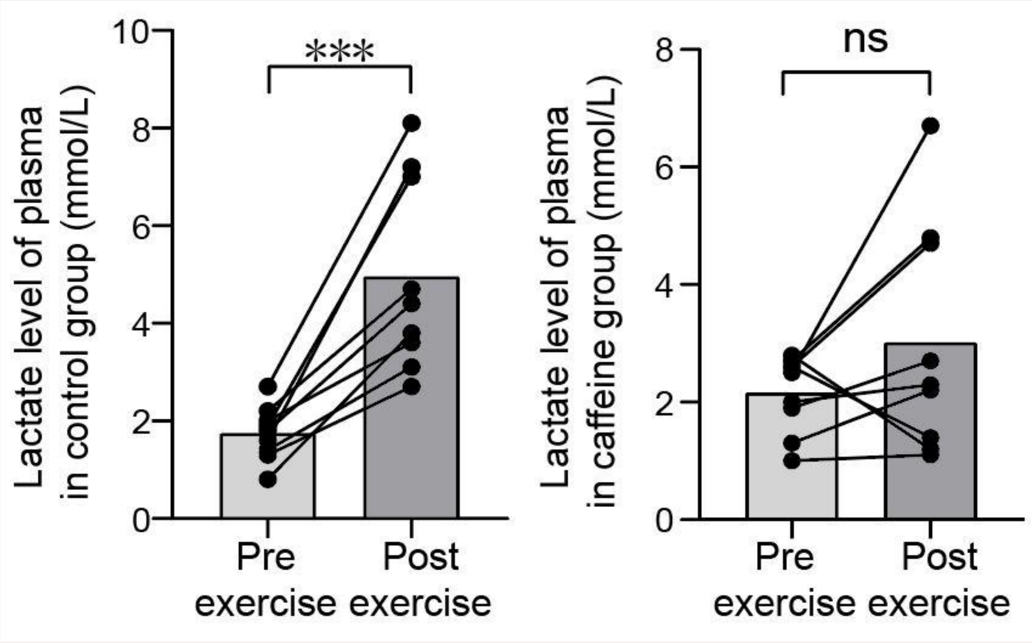

Conclusion: Caffeine significantly upregulates the expression of exercise-related proteins, and accelerates the utilization of lactate during exercise.
